# Blueberry Consumption and Changes in Obesity and Diabetes Mellitus Outcomes: A Systematic Review

**DOI:** 10.3390/metabo13010019

**Published:** 2022-12-22

**Authors:** Mayara Souza de Oliveira, Felipe Mateus Pellenz, Bianca Marmontel de Souza, Daisy Crispim

**Affiliations:** 1 Endocrinology Division, Hospital de Clínicas de Porto Alegre, Porto Alegre 90035-903, RS, Brazil; 2 Graduate Program in Medical Sciences: Endocrinology, Faculty of Medicine, Universidade Federal do Rio Grande do Sul, Porto Alegre 90010-150, RS, Brazil; 3 ULB Center for Diabetes Research, Medical Faculty, Université Libre de Bruxelles, 1070 Brussels, Belgium

**Keywords:** *Vaccinium* spp., blueberry, bilberry, obesity, metabolic syndrome, diet-induced obesity, systematic review

## Abstract

Low-grade inflammation and oxidative stress are key mechanisms involved in obesity and related disorders. Polyphenols from blueberry (BB) and bilberries (BiB) might protect against oxidative damage and inflammation. To summarize the effects of BiB or BB consumption in parameters related to obesity and its comorbidities, a search of the literature was performed in PubMed, Embase, and Cochrane Library repositories to identify all studies that evaluated associations of whole BB or BiB with obesity and associated disorders. Thirty-one studies were eligible for inclusion in this review: eight clinical trials and 23 animal studies. In humans, BB consumption only consistently decreased oxidative stress and improved endothelial function. In rodents, BB or BiB consumption caused positive effects on glucose tolerance, nuclear factor-kappa B (Nf-κb) activity, oxidative stress, and triglyceride (TG) content in the liver and hepatic steatosis. The high content of anthocyanins present in BB and BiB seems to attenuate oxidative stress. The decrease in oxidative stress may have a positive impact on glucose tolerance and endothelial function. Moreover, in rodents, these berries seem to protect against hepatic steatosis, through the decreased accumulation of hepatic TGs. BB and BiB might also attenuate inflammation by decreasing Nf-κb activity and immune cell recruitment into the adipose tissue.

## 1. Introduction

Obesity is a chronic disorder defined as abnormal body fat accumulation caused by an imbalance between energy intake and expenditure. Obesity prevalence has nearly triplicated since 1975 [1] and, nowadays, it affects about 22% of the people worldwide, constituting a global epidemic [2]. Obesity is a result of complex interactions among genetic, epigenetic, socioeconomic, cultural, and other environmental influences [3,4]. One of the main drivers of the obesity epidemic is the globalization of food systems, which produces more processed, affordable, and highly palatable food, and also promotes overconsumption of foods and beverages with high amounts of energy and low amounts of nutrients [5]. The reduction of physical activity due to changes in human lifestyle has also an important role in this epidemic [6].

The increased incidence of obesity leads to an increased risk of developing type 2 diabetes mellitus (T2DM), cardiovascular diseases, different types of cancers, and poor mental health [1]. T2DM is characterized by chronic hyperglycemia caused by a combination of insulin resistance (IR) and an inadequate compensatory insulin secretion, and usually occurs in subjects with obesity. The global prevalence of diabetes has reached 10.5% worldwide, with T2DM accounting for more than 90% of all cases [7]. An important link between obesity and its comorbidities, such as T2DM and IR, is the excessive production of reactive oxygen species (ROS), which is linked to the persistence of chronic low-grade inflammation, which characterizes the obesity state [8,9,10,11].

A number of studies have suggested that polyphenols and anthocyanins protect cells against oxidative damage and, therefore, reduce the risk of obesity [9], metabolic syndrome (MetS), T2DM, and IR [12,13,14]. Among all berries, the bilberries (BiB; *Vaccinium myrtillus*) and blueberries (BB; *Vaccinium* spp.) have the most diverse profile of anthocyanins, and BiB, specifically, has the highest content of anthocyanins [15]. Studies in humans have shown that consumption of BiB and BB can improve blood pressure (BP) [16], insulin sensitivity [17], and lipid metabolism [18,19,20,21], and it also reduces inflammatory [19,22] and oxidative stress [16,22] markers. Accordingly, in animal studies, BiB and BB consumption also seems to improve lipid [22,23,24,25] and glucose metabolisms [24,26,27] and reduce inflammation [22,28,29,30] and oxidative stress [28,29,31,32,33]. Moreover, consumption of these fruits appears to decrease body weight (BW) [27,34,35,36] and improve adipokine profiles, such as leptin [27,37], resistin, and retinol-binding protein 4 (RBP4) [38].

Even with these positive results, the effects of BB and BiB on obesity-related parameters are still inconclusive. Thus, this systematic review was performed to summarize the research findings on the effects of BB or BiB consumption on parameters associated with obesity and its comorbidities, including evidence from dietary intervention studies that used rodents or humans with MetS, obesity, or T2DM. An overview of possible mechanisms behind the berries’ effects on glucose tolerance, inflammation, and hepatic steatosis were also explored.

## 2. Material and Methods

This systematic review of the literature was conducted using the methodology outlined in the Cochrane Handbook for Systematic Reviews [39], and it was registered at PROSPERO (http:www.crd.york.ac.uk/PROSPERO) (accessed on 2 August 2022) under the CRD42021248091 number. Data are reported in line with the Preferred Reporting Items for Systematic Reviews and Meta-Analyses (PRISMA) [40]. Table S1 provides the PRISMA checklist.

### 2.1. Search Strategy and Eligibility Criteria

An electronic search of the literature was conducted in MEDLINE/PubMed, Cochrane Library, and EMBASE repositories to identify all studies that analyzed associations of BB or BiB consumption with obesity, MetS, or T2DM. The following medical subject headings (MeSH) were used: (“Blueberry” OR “Bilberry”) AND (“Obesity” OR “Diabetes” OR “Insulin resistance” OR “Metabolic syndrome X”). For the Cochrane Library search, the term “Vaccinium” was also included in order to amplify the identification of trials. The search was completed in August 2022, included human and animal studies, and was limited to papers written in English, Spanish, or Portuguese.

**Figure 1:**
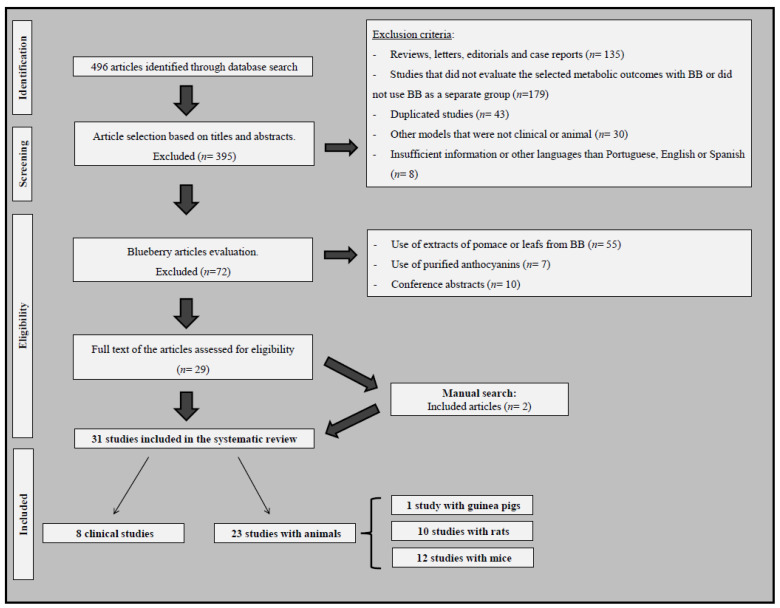
Flowchart illustrating the search strategy used in the systematic review.

We included clinical trials and intervention studies that evaluated the effects of consuming whole BB or BiB on metabolic parameters associated with obesity, T2DM, and MetS. Studies in humans or animals that used dietary interventions with BB or BiB (separated from other interventions) and analyzed biometric, metabolic, inflammatory, and oxidative stress parameters were included. Exclusion criteria were as follows: (1) reviews, letters, editorials, or case reports; (2) studies that did not evaluate the effect of BB or BiB on the outcomes of interest or did not analyze these berries in a separate group of patients or animals; (3) duplicated studies; (4) other models that were not clinical or animal (such as in vitro studies); and (5) insufficient information or other languages than Portuguese, English, or Spanish (Figure 1). In the case of duplicated results published more than once, we included the most complete article.

Two investigators (M.S.O. and F.M.P.) individually reviewed titles and abstracts of articles retrieved from the online repositories in order to evaluate which of them would be eligible for inclusion in this review, as described in previous systematic reviews from our group [41,42]. Divergences were settled by discussion between them and, when needed, a third reviewer (B.M.S.) was consulted. When abstracts did not provide enough information about the inclusion criteria, the full texts of the articles were read for evaluation. Articles that did not reach our eligibility criteria were excluded from the study. The reference lists from all articles fulfilling the eligibility criteria were also manually searched to identify other potentially important articles.

### 2.2. Data Extraction

Data were independently extracted by two researchers (M.S.O. and F.M.P.) applying a standard questionnaire, and agreement was sought in all extracted items [41,42]. In the case of an agreement could not be reached, differences during the data extraction were resolved by a third reviewer (B.M.S.) or reading the original article. Data extracted from each human or animal study were as follows: (1) characteristics of the study (first author, publication year, and study design); (2) sample description (clinical or animal model, sample sizes, population or rodent breed); (3) characteristics of subjects [age, body mass index (BMI), BW before and after the intervention, and analyzed tissues]; (4) characteristics of the dietary intervention (type and processing of fruits, dose/concentration); and (5) analyzed outcomes, such as gene or protein expressions, laboratory parameters (lipid and glycemic profiles, and cytokine/chemokine levels), and biometric and body composition information, among others.

## 3. Results

### 3.1. The Literature Search and Characteristics of Eligible Studies

Figure 1 shows the strategy followed to identify and select studies for inclusion in the systematic review. A total of 496 possibly relevant articles were retrieved by searching electronic databases, and 395 of them were excluded during the review of titles and abstracts, since they did not fulfill our inclusion criteria.

One hundred and one articles, therefore, appeared to be eligible at this point and had their full texts evaluated. However, after full-text analysis, 72 articles were excluded because of the use of pomace or leaf extracts instead of the whole fruit, as well as the use of purified anthocyanins or conference abstracts. Therefore, a total of 29 articles fulfilled the eligibility criteria [16,17,18,19,20,21,22,23,24,26,27,28,29,30,32,33,35,36,37,38,43,44,45,46,47,48,49,50,51]. Moreover, two articles were identified by a manual search of the reference lists of the 29 articles [31,34], totalizing 31 articles. Among the included studies, eight were clinical trials [16,17,18,19,22,43,46,47], 10 used animal models with rats [21,23,26,28,30,32,33,35,38,49], 12 were animal models with mice [20,24,27,29,34,36,37,44,45,48,50,51], and one study included Guinea pigs [31].

Clinical studies comprised patients with MetS [16,18,19,22,43,47], T2DM [46] and IR [17], and the follow-up ranged from 24 h to 6 months. Studies with rodent models evaluated the effects of BB or BiB consumption in diet-induced obesity [20,27,28,29,31,33,34,35,36,37,44,45,48,49,50,51], genetic models of obesity [21,23,26,30,32,38], and menopause-induced obesity [24]. In animals, the follow-up ranged from 72 days to 15 weeks, with most of the studies having an eight-week follow-up.

Regarding the way of offering the fruit, most of the studies offered freeze-dried powdered BB or BiB [16,17,20,22,23,24,26,27,28,29,30,31,32,34,35,36,38,43,44,46,47,48,50,51,52,53]. Fruits were also offered in the format of extruded and unextruded BB or BiB pomace [49], juice [33,37], fresh fruits alone [18], or mixed with freeze-dried fruits [19]. In this review, we focused on results using fresh and freeze-dried fruits to evaluate the effect of whole fruit instead of its specific components. Considering the fruit species, studies used *Vaccinium* (*V*.) *myrtillus* [19,23,34,35,36,51], *V*. *angustifolium* [21,30], *V*. *ashei* [24], *V*. *corymbosum* L. [27,31,33], or the combination of *V*. *ashei* and *V*. *corymbosum* [26,28,29,48,50], *V*. *angustifolium* and *V*. *corymbosum* [16], *V*. *myrtillus* and *V*. *corymbosum* [17,47], *V*. *corymbosum,* and *V*. *virgatum* [46].

### 3.2. Clinical Studies That Evaluated the Effects of Blueberry or Bilberry Consumption on Obesity-, MetS- or T2DM-Related Outcomes

The clinical studies regarding BB or BiB treatment in humans are described in Table 1. Detailed information on patient characteristics, inclusion and exclusion criteria, and other relevant information can be found in Table S2. The most analyzed parameters in these studies were lipid profile, inflammatory markers, BP and endothelial function, oxidative stress, glycemic control and insulin sensitivity/IR, and BW and other biometric parameters. The results of these studies are summarized in Table 2 and are described below.

Lipid profile was evaluated in seven studies [16,17,18,19,43,46,47]. Regarding triglycerides (TGs), five studies showed no differences between BB or BiB and control groups in patients with MetS [16,19,43,47] or obesity and IR [17]. In contrast, one study in T2DM patients showed decreased TG levels [46], while another study with MetS patients reported increased TG levels in the BB group (vs. controls) [18]. Regarding total cholesterol levels, six studies did not observe any difference between MetS group or patients with obesity from treated or non-treated groups [16,17,18,19,46,47], while one study showed decreased total cholesterol levels in patients with MetS treated with BB (vs. controls) [43]. All seven studies that evaluated LDL levels did not show any difference between treated and non-treated groups [16,17,18,19,43,46,47]. Two studies in MetS patients showed increased HDL levels in the BB group (vs. controls) [18,43], while five others did not observe any difference in HDL between groups [16,17,19,46,47].

Inflammation markers were evaluated in five studies [16,17,19,22,46]. One study in patients with MetS reported an increased number of myeloid dendritic cells and decreased monocyte expression of inflammatory cytokines [(tumor necrosis factor (*TNF*) and interleukin (*IL*)-6], toll-like receptor 4, and serum granulocyte-macrophage colony-stimulating factor in the BB group (vs. controls) [22]. Accordingly, another study showed a decrease in the total inflammation score, IL6, IL12, C-C motif chemokine receptor 2, and high sensitivity C-reactive protein (hs-CRP) levels in adults with MetS who consumed BiB (vs. controls) [19]. However, three other studies did not find significant changes in inflammatory parameters between treated and non-treated groups [16,17,46] (Table 2). Moreover, two studies evaluated adipokine levels. Kolehmainen et al. [19] did not observe any differences in leptin and adiponectin levels between patients with MetS from treated and non-treated groups. Accordingly, Basu et al. [16] reported similar adiponectin levels between MetS groups.

Regarding endothelial function and BP, three studies observed an improvement in endothelial function in individuals with MetS who consumed BB (vs. controls) [18,43,47]. A reduction in BP was reported by one study in subjects with MetS who consumed BB (vs. controls) [16]. However, five other studies did not find any difference in BP between groups [17,18,43,46,47].

Two studies suggested that BB treatment decreased oxidative stress markers. Basu et al. [16] observed a decrease in serum lipid peroxidation products [malondialdehyde (MDA) and hydroxynonenal (HNE)] in adults with MetS who consumed BB (vs. controls). Nair et al. [22] reported a decrease in superoxide and total ROS in blood and monocytes from subjects with MetS who consumed BB (vs. controls).

Among the seven studies that analyzed glycemic outcomes, five did not show any differences between fasting plasma glucose (FPG) or glycated hemoglobin (HbA1c) between patients from the treated and non-treated groups [16,17,18,19,47]. However, one study in patients with MetS showed a decrease in postprandial (24 h) glucose in the BB group (vs. controls) [43]. Another study in men with T2DM showed a decrease in HbA1c values after BB treatment even though FPG levels did not differ between groups [46]. In addition, two studies reported improved glucose tolerance [decreased area under the curve (AUC) of glucose during glucose tolerance test (GTT)] in the BB group (vs. controls) [17,47]. Four studies showed no differences in insulin levels or IR between BB groups and patients who did not consume this fruit [16,17,18,47]. In contrast, Curtis et al. [43] reported decreased insulin levels in patients with MetS who consumed BB, while Stull et al. [17] showed that BB treatment improved insulin sensitivity in patients with obesity and IR (vs. controls). Moreover, BW and related anthropometric parameters [fat mass (FM) and fat-free mass (FFM) did not differ between patients from the treated and non-treated groups [16,17,19,46,47].

### 3.3. Studies with Rodent Models That Evaluated the Effects of Blueberry or Bilberry Consumption on Obesity-, MetS- or T2DM-Related Outcomes

All animal studies that have evaluated the effect of BB or BiB consumption in the outcomes of interest are shown in Table 3. In general, these studies suggested that glucose tolerance, nuclear factor-kappa B (Nf-κb) related-inflammation, oxidative stress, hepatic steatosis, and TG content in the liver are the main parameters affected by BB or BiB diet (Table 2).

Lipid profile was evaluated in 12 studies [21,23,24,26,31,33,34,35,37,48,49,51]. Six studies reported a decrease in total cholesterol levels in animals treated with BB or BiB diet (vs. controls) [21,23,31,34,35,49], while five other studies did not find any difference between experimental groups [26,34,37,48,51]. Although Heyman et al. [34] did not observe any difference in serum cholesterol levels in treated and non-treated mice, they showed a decrease in cholesterol levels in the liver of animals from the BiB group. Regarding HDL, four studies did not find any difference in HDL levels between study groups [21,26,31,34]; however, one study reported decreased HDL levels in rats fed the BiB diet (vs. controls) [23]. Moreover, two studies showed decreased LDL levels in rats treated with BiB or BB (vs. controls) [23,31], while one study reported similar LDL levels between study groups [34]. Five studies reported that BB or BiB treatment caused a reduction in serum/plasma TG levels [21,24,26,33,49]. However, five other studies did not find any difference in TG levels between animals fed with a BB or BiB diet and controls [23,31,34,35,37].

Despite inconclusive results about serum/plasma TG levels, four studies showed that BB or BiB consumption decreased hepatic TG content in animals (vs. controls) [24,31,33,34]. Two other studies did not find any difference in hepatic TG content between experimental groups [23,48]. Moreover, four studies showed a decrease in hepatic steatosis [24,33,35] and hepatic enzymes [alanine aminotransferase (ALT) and aspartate aminotransferase (AST)] [31] in animals fed with BB or BiB (vs. controls). In contrast, Morissette et al. [48] did not observe any effect of the BB treatment on AST and ALT levels in mice.

Sixteen studies evaluated inflammation-associated parameters [23,24,26,27,28,29,30,32,33,37,38,45,48,49,50,51]. Data concerning cytokine and chemokine expressions are mostly inconclusive. Five studies reported a decrease in Tnf (gene or protein) [28,29,30], Il6 [30], *Il10* [29], *Il1β* [28,32], *Il18* [32], transforming growth factor-β [32], and CC motif chemokine ligand 2 (Ccl2/Mcp1) [51] expressions in animals fed with a BB or BiB diet (vs. controls). In contrast, six studies showed no differences in Tnf [26,33,48,51], Il6 [26,28,29,48,51], Il1β [33,51], Il12 [48,51], *Interferon-γ* (Ifnγ) [33,48], Il7 [51], *Il10* [33], *Ccl2* [29,48], and CC motif chemokine ligand 5 [48] levels between experimental groups.

Regarding transcription factors and other proteins related to inflammatory and immune responses, three studies reported that BB consumption induced a decrease in Nf-κb activity (vs. controls) [28,30,32], while Nunes et al. [33] did not observe any difference in Nf-κb and signal transducer and activator of transcription 3 activities between groups. *C-jun N-terminal kinase* expression [23] and p38-mitogen-activated protein kinase phosphorylation [32] were decreased in mice from the BB group (vs. controls). Moreover, a decrease in the recruitment of pro-inflammatory M1 macrophages to the adipose tissue [28,29], type 1 inflammatory response [natural killer cells, T helper (Th) 1, Th1/Th2, and Ifnγ-producing T cells) [51], and toll-like receptor 4 expression [32] was observed in mice fed a BB or BiB diet (vs. controls). Lewis et al. [50] also reported that supplementation of high-fat diet (HFD) with BB attenuated the HFD-associated reduction in ex vivo T cell proliferation in splenocytes.

Regarding adipokines, three studies showed a reduction in leptin [27,37,45] and two in resistin [38,51] levels in animals from the fruit group (vs. controls). However, three other studies did not show any difference in leptin levels between groups [29,49,51]. Vendrame et al. [30] showed increased adiponectin levels in rats from the BB group (vs. controls), which was in contrast with five other studies that reported similar adiponectin levels between groups [23,24,29,45,51].

Five studies evaluated parameters related to oxidative stress. Four of them showed a reduction in markers of oxidative stress (MDA, superoxide, and peroxynitrite) in animals from the BB group [28,31,32,33], while one study showed similar levels of thiobarbituric acid reactive substances (TBARS) in rats fed with BB (vs. controls) [48]. Moreover, four studies reported an increase in the antioxidant enzyme levels in the fruit group (vs. controls) [29,31,32,33], while one study did not find any differences in glutathione (GSH), glutathione peroxidase (GSH-Px), and superoxide dismutase (SOD) levels between groups [31]. Regarding BP, two studies reported decreased BP values in mice fed with BB or BiB (vs. controls) [32,51], while two others did not find any difference between groups [23,36].

In relation to the glycemic profile, 16 studies evaluated fasting blood glucose and/or HbA1c levels. Most of them showed similar blood glucose or HbA1c levels between groups [20,23,26,28,33,34,36,37,38,44,48,49]. Four studies showed decreased blood glucose levels in animals fed with BB or BiB [23,24,29,51], and only one study reported decreased HbA1c levels in rats treated with BB (vs. controls) [38]. Fourteen studies analyzed glucose response after a GTT. Ten of them showed improved glucose tolerance [decreased AUC_glucose_ after GTT] in animals from the fruit group compared to controls [20,24,26,27,28,29,32,33,44,51], while four studies did not observe any difference in glucose tolerance between groups [23,44,48,49].

Concerning insulin levels, four studies reported a decrease in insulin levels in mice and rats fed a BB or BiB diet (vs. controls) [20,26,34,49]. In contrast, seven studies observed similar insulin levels between experimental groups [23,28,29,33,37,38,48]. Moreover, seven studies reported decreased IR in animals from the fruit group [20,26,28,29,33,34,49], while five other studies did not observe any reduction in IR in the treated group (vs. controls) [23,37,38,48,51]. Interestingly, Brader et al. [23] reported decreased glucose transporter (Glut) *2* and insulin receptor substrate 1 (Irs1) expressions in the liver and increased *Glut4* expression in adipose tissue of rats fed with BiB compared to the control group. Seymour et al. [26] observed increased *Glut4* and *Irs1* expressions in both adipose tissue and skeletal muscle of rats fed with BB, as well as increased *Ucp3* expression in the muscle (vs. controls).

A total of 22 studies evaluated biometric parameters. Two studies reported an increase in BW [44,50], while four studies reported a decrease in BW or delta (Δ)-BW in animals from the berry groups (vs. controls) [27,34,35,51]. However, another 17 studies did not find any differences in BW or Δ-BW between groups [20,21,23,24,26,28,29,30,32,33,36,37,38,45,48,49,51]. Two studies observed a reduction in FM [34,45], while two others reported an increase in this parameter [44,50] in mice and rats fed a BB or BiB diet compared to controls. In contrast, eight studies did not find any difference in FM between experimental groups [24,26,28,29,37,48,49,51]. Two studies reported an increase in FFM in mice fed with BB (vs. controls) [44,45]; however, five studies did not observe any difference in FFM between groups [24,26,34,37,48]. Although Liu et al. [35] did not observe any difference in BW between experimental groups, they observed an increased interscapular brown adipose tissue mass and *uncoupling protein 1* expression in rats fed with BiB compared to controls.

## 4. Discussion

In the last decades, global dietary patterns have changed to an increase in non-healthy diets, which are directly associated with the progressive growth of obesity and T2DM prevalence. It is well known that healthy diet modifications, such as the inclusion of fruits and vegetables, can ameliorate and even retard the harmful effects caused by these diseases. In this review, we summarized the available data on the effect of whole BB or BiB consumption in the different parameters associated with obesity, T2DM, and MetS. Evidence presented here indicates the benefits of BB and/or BiB consumption in reducing oxidative stress, hepatic steatosis, and NF-κB-related inflammation, and also in improving glucose tolerance, mainly in rodents. In humans, BB consumption seems to have a positive impact in decreasing oxidative stress and improving endothelial function.

BB and BiB are blue-colored fruits that belong to the genus *Vaccinium*, family *Ericaceae*. The main functional components of these berries are anthocyanins, known to be powerful natural antioxidants. Regular consumption of darker-colored berries may provide a high intake of anthocyanins and other phenolic compounds [54,55,56]. BB and BiB are also rich sources of a wide variety of nutritive compounds, including sugars (glucose, fructose) and minerals (phosphorus, calcium, iron, potassium, magnesium, manganese, sodium, and copper) [54]. Usually, berries contain a large amount of vitamins A, C, and E, which act as antioxidants and may reduce the inflammatory process and oxidative stress [57]. It is noteworthy that no unwanted or toxic effects have been associated with the consumption of BB [54,58]. Cladis et al. [59] observed that purified polyphenols from BB had no adverse effect in rats when in a concentration equivalent to ~10 g BB polyphenols/per day for a 70 kg human.

Moreover, BB and BiB contain low concentrations of lipids but high concentrations of fermentable dietary fibers, which have a nutritional important function and reduce serum LDL levels [54]. Moreover, high concentrations of anthocyanins have been found in the distal intestine, where they can interact with the gut microbiota, being recognized as a prebiotic food. Hence, BB and BiB might improve obesity-related inflammation and oxidative stress by altering the gut microbiota composition [35,48,60]. Of note, greater beneficial effects have been associated with the antioxidants obtained from whole foods than those obtained from stand-alone dietary supplements [55,61,62]. For these reasons, this review was focused on studies that analyzed whole fruits instead of their purified compounds.

Oxidative stress can be identified by different biomarkers, including markers of lipid peroxidation, such as MDA, TBARS, and HNE, conjugated dienes or F2-isoprostanes, as well as levels of antioxidant enzymes, such as SOD, catalase, and GSH-Px, among others [54]. Previous studies have demonstrated that the consumption of berries rich in antioxidant phenolic compounds results in an increase in plasma total antioxidant status in humans (reviewed in [54]). The antioxidant effects of BB have also been shown by studies using cellular and animal models of oxidative stress [63,64], reinforcing the role of this fruit as an antioxidant dietary supplement. In the context of obesity and T2DM, it is well known that both glucotoxicity and lipotoxicity are key drivers of oxidative stress and inflammation [65]; thus, the improvement of the systemic antioxidant status may have beneficial effects on these diseases. Here, our systematic review indicates that BB or BiB consumption is able to decrease oxidative stress markers [28,31,32,33] and increase antioxidant enzyme levels in both humans and rodents [29,31,32,33].

**Figure 2:**
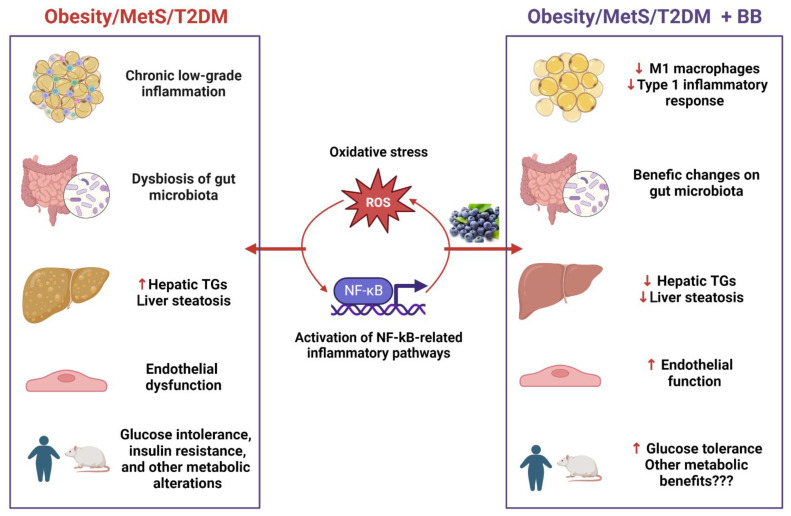
The effects of blueberries (BB) and bilberries in outcomes related to obesity, metabolic syndrome (MetS), and type 2 diabetes mellitus (T2DM). Obesity and associated metabolic disorders are characterized by systemic and chronic low-grade inflammation, dysbiosis of the gut microbiota, and other metabolic alterations, including increased triglycerides (TGs) content in the liver, liver steatosis, glucose intolerance, insulin resistance, and endothelial dysfunction. The main mechanisms behind these alterations are the increased oxidative stress and activation of the nuclear factor-κB (NF-κB)-related pro-inflammatory pathways. Anthocyanins and other components of blueberries and bilberries are able to decrease the production of reactive oxygen species (ROS) and the activation of the transcription factor NF-κB, thus decreasing the expression of its pro-inflammatory target genes. The attenuation of these two key mechanisms may lead to: (1) decreased recruitment of pro-inflammatory M1 macrophages into the adipose tissue and type 1 inflammatory response (T helper cells); (2) benefic changes on the composition of gut microbiota, further decreasing NF-κB activation; (3) decreased TG content in the liver and protection against hepatic steatosis; (4) improvement of endothelial function; and (5) improvement of glucose tolerance. Other beneficial effects of BB consumption still need to be confirmed by well-designed clinical studies. ↓ Reduction; ↑ Increase. This figure was created using BioRender.com (accessed on 1 December 2022).

An important characteristic of obesity is a low-grade inflammation, which is associated with the infiltration of monocytes and other immune cells into the adipose tissue, leading to metabolic abnormalities [22]. Inflammation and oxidative stress are strongly interconnected, so inflammation increases ROS production, and oxidative stress triggers inflammatory factors [66]. Thus, by decreasing ROS production, anthocyanins might also decrease inflammation (Figure 2). However, our systematic review shows that the studies available today do not support a significant effect of BB or BiB consumption on cytokine, chemokine, or adipokine levels. It is important to mention that there are only a small number of studies that analyzed specific inflammatory markers in different tissues.

Despite the inconclusive results regarding cytokines and other inflammatory markers, it seems that BB consumption is able to decrease Nf-κb activity in rodents [28,30,32]. NF-κB is a key transcription factor that regulates cellular immune responses to infection and oxidative stress by activating pro-inflammatory pathways [30,67,68]. In agreement with the studies using whole berries, Huang et al. [69] reported that purified anthocyanins from BB had anti-inflammatory properties by attenuating the NF-κB pathway in endothelial cells, consequently decreasing the expression of *TNF*, *MCP1*, intercellular adhesion molecule 1, and vascular cell adhesion molecule 1, which are associated with mononuclear cell infiltration. Other studies also show that purified anthocyanins increase nitric oxide bioavailability and inhibit peroxynitrite-induced NF-κB activation [70,71,72,73]. Although the mechanisms by which anthocyanins inhibit NF-κB activation are not well understood, these molecules might act as redox buffers able to suppress oxidative stress and thereby dampen the inflammatory response by direct ROS scavenging [73]. Moreover, it is well known that dysbiosis of the gut microbiota can lead to the translocation of bacterial pro-inflammatory factors into the circulation, activating the NF-κB (Figure 2). Interestingly, Lee et al. [28] reported that BB treatment decreased Nf-κb-related inflammation through a benefic alteration of gut microbiota composition in rats.

In line with the effect of anthocyanins in inhibiting NF-κB-related inflammation, it seems that BB or BiB consumption can also decrease the recruitment of pro-inflammatory M1 macrophages into the adipose tissue [28,29], as well as the type 1 inflammatory response [51] (Figure 2). The beneficial effects of monocyte modulation have been observed in both clinical and animal studies [17,19,22,29,51]. Nair et al. [22] showed that attenuation of oxidative stress in monocytes after BB consumption decreased pro-inflammatory markers and increased the number of myeloid dendritic cells, thereby regulating the inflammatory balance.

Attenuation of oxidative stress and inflammation may have beneficial effects on endothelial function and glucose metabolism, possibly protecting against cardiometabolic diseases [74]. Accordingly, BB consumption seems to improve endothelial function in humans [18,43,47]. In vitro studies suggest that anthocyanins may ameliorate endothelial function by decreasing oxidative stress and increasing nitric oxide synthase levels in endothelial cells, consequently decreasing vasoconstriction via a nitric oxide-mediated pathway [75,76,77]. This improvement in endothelial function might decrease BP [16,32,51], although this beneficial effect was not confirmed by other studies in humans and rodents [17,18,23,36,43,46,47]. Of note, many patients included in the clinical studies were using antihypertensive medications (Table S2), which may have lessened the clinical beneficial effects of BB and BiB on BP.

Despite having no confirmed effects on fasting blood glucose and insulin levels, BB or BiB consumption seems to improve glucose tolerance in rodents [20,24,26,27,28,29,32,33,44,51] and humans [17,47]. Liu et al. [20] suggested that BB improves glucose tolerance by enhancing pancreatic beta-cell survival and decreasing the expression of pro-inflammatory cytokines and oxidative stress. Upregulation of the *Glut2* and *Glut4* expressions [23,26], activation of peroxisome proliferator-activated receptor-γ (PPARγ) and AMP-activated protein kinases, and downregulation of *RBP4* expression [78] may also contribute to the BB-induced improvement in glucose tolerance. Moreover, glucose tolerance might be positively affected by the modulation of gut microbiota after BB consumption [48]. Inconclusive results regarding glucose and insulin levels and IR might be explained by different study follow-ups, patients with different clinical characteristics, as well as different rodent strains, fasting times, and the way of offering the fruits. Thus, the effect of BB or BiB on glucose metabolism must be confirmed in additional well-designed clinical trials.

Many patients who are overweight and who suffer from obesity also suffer from non-alcoholic fatty liver disease (NAFLD), which is a spectrum of conditions characterized by macrovesicular steatosis of the liver. It ranges from simple fatty liver (steatosis) to non-alcoholic steatohepatitis, which can lead to end-stage liver disease. NAFLD is associated with increased inflammation, oxidative damage, and impaired insulin sensitivity [79]. Therefore, decreasing lipid accumulation in the liver may protect against NAFLD. Here, despite the inconclusive results on serum/plasma cholesterol and TG levels, we observed that BB or BiB consumption is able to decrease the hepatic TG content in rodents [24,31,33,34], thus protecting against hepatic steatosis [24,31,33,35]. According to Elks et al. [24], a possible explanation for the prevention of hepatic steatosis is an enhancement in hepatic fatty acid oxidation. Both in vitro and in vivo studies have shown that anthocyanins regulate the expression of key enzymes involved in TG and cholesterol metabolism, including lipoprotein lipase, fatty acid synthase, ATP-binding cassette transporter 1, PPARγ, and sterol regulatory element binding protein 1 [21,24,80,81,82].

There are a few limitations that should be considered in this review. First, due to the fact that we only included papers in English or Spanish, we might have missed some evidence in other languages. Second, the selection of only whole fruits could be a limitation since there are many studies showing the beneficial roles of anthocyanins extracted from BB or BiB on the outcomes of interest. Third, the studies evaluated in this review differ in many aspects in terms of their methodologies, including different follow-up periods, patients with different characteristics and disorders, different rodent species and strains, fasting times for laboratory analysis, and the way of offering the fruits, which can partially explain the inconclusive results.

## 5. Conclusions

In this review, we showed that BB and BiB may have beneficial roles on obesity-, MetS- and T2DM-related outcomes, mainly due to positive effects in glucose tolerance, endothelial function, hepatic steatosis, and decreased NF-kB activity. The main mechanism behind the benefic effects of BB and BiB on obesity- and T2DM-related outcomes is the attenuation of oxidative stress, which consequently dampens Nf-kB pro-inflammatory actions and improves the composition of the gut microbiota. Based on these results, BB and BiB may be considered as natural adjuvant supplements to reduce inflammation and oxidative stress and improve glucose metabolism in patients with obesity, MetS, and/or T2DM. However, considering that the studies included in this systematic review differ in many methodological aspects, which is a limitation of the available literature, it is essential to perform more well-designed clinical trials to better understand if the anti-inflammatory effects observed in rodents are confirmed in humans and to elucidate how the connection between anthocyanins and pro-inflammatory NF-κB, fatty acid oxidation, as well as IR pathways, occurs. Moreover, it is necessary to evaluate the ideal amount of BB and BiB to improve obesity-related outcomes in humans considering both safety and calories.

## Figures and Tables

**Table 1:** Clinical studies that evaluated the effect of blueberry or bilberry consumption on obesity and related-metabolic parameters.

1st Author, Year (Ref)	Sample	Blueberry Treatment	Follow-Up	Results (BB vs. Placebo/Control Group)
Basu, 2010 [16]	48 subjects with MetS: BB group (*n* = 25) vs. controls (*n* = 23)	50 g freeze-dried BB (*V. angustifolium/V. corymbosum* L.) in a beverage, daily	8 weeks	↔ No differences in BW, WC, HbA1c, HOMA-IR, FPG, lipid profile, IL6, adiponectin, and hs-CRP; ↓ BP, oxidized LDL, and lipid peroxidation (MDA and HNE);
Curtis, 2019 [18]	115 subjects with MetS: 150 g BB (*n* = 37), 75 g BB (*n* = 39) vs. controls (*n* = 39)	75 g or 150 g fresh BB	6 months	↔ No differences in BP, TC, LDL, FPG, HbA1c, insulin, and IR; ↑ HDL (150 g) and TGs (75 g); improved endothelial function (150 g);
Curtis, 2022 [43]	45 subjects with MetS: BB (*n* = 23) vs. controls (*n* = 22)	26 g of freeze-dried BB (= 150 g of fresh BB)	24 h	↔ TG and LDL, BP and Apo-B; ↓ Postprandial glucose (0–24 h), insulin, total cholesterol, and Apo-A1; ↑ HDL; improved endothelial function;
Kolehmainen, 2012 [19]	27 subjects with MetS: BiB diet (*n* = 15) vs. controls (*n* = 12)	400 g fresh BiB [200 g BiB puree + 40 g dried BiB (= 200 g fresh BiB; *V. myrtillus*), daily	8 weeks	↔ No differences in BW, lipid profile, FPG, adiponectin and leptin; ↓ inflammation score, hs-CRP, IL6, and IL12, expression of *MMD* and *CCR2* (monocyte and macrophage function) in PBMCs;
Nair, 2017 [22]	27 subjects with MetS: BB (*n* = 15) vs. controls (*n* = 12)	22.5 g freeze-dried BB, twice a day	6 weeks	↓ Superoxide and total ROS (blood and monocytes), *TNF*, *IL6* and *TLR4* expressions (monocytes), and GMCSF (serum inflammatory marker); ↑ myeloid dendritic cells;
Stote, 2020 [46]	52 men with T2DM: BB (*n* = 26) vs. placebo (*n* = 26)	22 g freeze dried BB, daily (*V. virgatum*/*V. corymbosum*)		↔ No differences in FPG, insulin, total cholesterol, LDL, HDL, and hs-CRP, BP, and BW; ↓ HbA1c, fructosamine, TG, AST, and ALT;
Stull, 2010 [17]	32 individuals with obesity and IR: BB (*n* = 15) vs. controls (*n* = 17)	22.5 g freeze-dried BB in a smoothie (*V. myrtillus/V. corymbosum*, 1:1), twice a day (45 g/day)	6 weeks	↔ No differences in BW, BMI, FFM, FM, inflammatory markers (hs-CRP, TNF and MCP-1), lipid profile, insulin, and BP; ↑ insulin sensitivity;
Stull, 2015 [47]	44 individuals with MetS: BB (*n* = 23) vs. control (*n* = 21)	22.5 g freeze-dried BB in a smoothie (*V. myrtillus/V. corymbosum*, 1:1), twice a day (45 g/day)	6 weeks	↔ No differences in BW, BMI, FFM, FM, BP, FPG, insulin, and lipid profile; improved endothelial function.

↓ Decreased values; ↑ Increased values; Apo: apoliprotein; HbA1c: glycated hemoglobin; ALT: alanine aminotransferase; AST: aspartate aminotransferase; BB: blueberry; BiB: bilberry; BMI: body mass index; BP: blood pressure; BW: body weight; CCR2: C-C chemokine receptor 2; FFM: fat free mass; FM: fat mass; FPG: fasting plasma glucose; GMCSF: granulocyte macrophage colony-stimulating factor; HNE: hydroxynonenal (lipid peroxidation marker); HOMA-IR: homeostatic model assessment for insulin resistance; hs-CRP: high-sensitive *C*-reactive protein; IL6: interleukin 6; MCP-1: monocyte chemoattractant protein-1; MDA: malondialdehyde (lipid peroxidation marker); MetS: metabolic syndrome; MMD: monocyte to macrophage differentiation associated; PBMCs: peripheral blood mononuclear cells; ROS: reactive oxygen species; TC: total cholesterol; TNF: tumor necrosis factor; TLR4: toll-like receptor 4; T2DM: type 2 diabetes mellitus; TGs: triglycerides; *V*.: *Vaccinium*; WC, waist circumference.

**Table 2:** Summary of the main results of studies that have evaluated the effect of blueberry or bilberry consumption in obesity and related-metabolic parameters in human and rodents.

Outcomes	Human	Mouse, Rat, or Guinea Pig	Conclusion
Lipid Profile			
TGs (plasma/serum)	↔ 5 studies [16,17,19,43,47] ↑ One study [18] ↓ One study [46]	↔ 5 studies [23,31,34,35,37] ↓ 5 studies [21,24,26,33,49]	↔ Humans Inconclusive in rodents
Hepatic TG content	No study	↔ Two studies [23,48] ↓ 4 studies [24,31,33,34]	↓ Rodents
Total cholesterol	↔ 6 studies [16,17,18,19,46,47] ↓ One study [43]	↔ 5 studies [26,34,37,48,51] ↓ 6 studies [21,23,31,35,49]/↓ liver [34]	↔ Humans Inconclusive in rodents
LDL	↔ 7 studies [16,17,18,19,43,46,47]	↔ One study [34] ↓ 2 studies [23,31]	↔ Humans Inconclusive in rodents
HDL	↔ 5 studies [16,17,19,46,47] ↑ 2 studies [18,43]	↔ 4 studies [21,26,31,34] ↓ One study [23]	↔ In general
Hepatic steatosis and liver enzymes	↓ ALT/AST [46]	↓ Steatosis—3 studies [24,33,35] ↓ ALT/AST [31] ↔ ALT/AST [48]	Inconclusive in humans ↓ Rodents
Inflammatory markers			
IL-1β	No study	↔ 2 studies [33,51] ↓ 2 studies [28,32]	Inconclusive in general
IL-6	↔ One study [16] ↓ 2 studies [19,22]	↔ 5 studies [26,28,29,48,51] ↓ One study [30]	Inconclusive in humans ↔ Rodents
TNF	↔ One study [17] ↓ One study [22]	↔ 4 studies [26,33,48,51] ↓ 3 studies [28,29,30]	Inconclusive in general
Nf-kb	No study	↓ 3 studies [28,30,32] ↔ One study [33]	↓ Rodents
Leptin (pro-inflammatory)	↔ One study [19]	↔ 3 studies [29,49,51] ↓ 3 studies [27,37,45]	Inconclusive in general
Adiponectin (anti-inflammatory)	↔ 2 studies [16,19]	↔ 5 studies [23,24,29,45,51] ↑ One study [30]	↔ In general
BP and endothelial function			
BP	↔ 5 studies [17,18,43,46,47] ↓ One study [16]	↔ 2 studies [23,36] ↓ 2 studies [32,51]	↔ Humans Inconclusive in rodents
Endothelial function	↑ 3 studies [18,43,47]	No study	↑ Humans
Oxidative stress and antioxidant status			
Oxidative stress markers	↓ 2 studies—MDA and HNE [16], total ROS and superoxide [22]	↔ One study: TBARS [48] ↓ 4 studies: MDA [28,31,33], ROS, superoxide, and peroxynitrite [32]	↓ In general
Antioxidant enzymes	No study	↔ One study: GSH, GSH-Px, SOD [31] ↑ 4 studies: GST [31], GSH-Px [29], GSH [33], SOD and catalase [32]	↑ In rodents
Glycemic profile and insulin sensitivity			
Glucose (FBG, FPG or postprandial glucose levels)	↔ 6 studies [16,17,18,19,46,47] ↓ One study [43]	↔ 12 studies [20,23,26,28,33,34,36,37,38,44,48,49] ↓ 5 studies: fasting [23,24,29,51] * and fed [33]	↔ In general
HbA1c	↔ 2 studies [16,18] ↓ One study [46]	↔ One study [23] ↓ One study [38]	Inconclusive in general
Glucose tolerance (↓ AUC_glucose_ GTT)	↑ 2 studies [17,47]	↔ 4 studies [23,44,48,49] ↑ 10 studies [20,24,26,27,28,29,32,33,44,51]	↑ In general
Insulin resistance (HOMA-IR or ITT)	↔ 2 studies [16,18] ↓ One study [17]	↔ 5 studies [23,37,38,48,51] ↓ 7 studies [20,26,28,29,33,34,49]	Inconclusive in general
Insulin levels	↔ 3 studies [17,18,47] ↓ One study [43]	↔ 7 studies [23,28,29,33,37,38,48] ↓ 4 studies [20,26,34,49]	↔ Humans Inconclusive in rodents
Body measures			
Body weight, BMI, or weight gain	↔ 5 studies [16,17,19,46,47]	↔ 17 studies [20,21,23,24,26,28,29,30,32,33,36,37,38,45,48,49,51] ↑ 2 studies [44,50] ↓ 4 studies [27,34,35,51]	↔ In general
Fat mass	↔ 2 studies [17,47]	↔ 8 studies [24,26,28,29,37,48,49,51] ↑ 2 studies [44,50] ↓ 2 studies [34,45]	↔ In general
Fat-free mass	↔ 2 studies [17,47]	↔ 5 studies [24,26,34,37,48] ↑ 2 studies [44,45]	↔ In general

* Glucose: Brader et al. [23] showed similar fasting plasma glucose (FPG) between groups, but a decrease in the whole blood glucose in the bilberry group. ↔ Similar values between groups; ↓ Decreased values; ↑ Increased values; ALT: alanine aminotransferase; AST: aspartate aminotransferase; AUC_glucose_ GTT: area under the curve of glucose in the glucose tolerance test; BP: blood pressure; FBG: fasting blood glucose; FPG: fasting plasma glucose; GSH: glutathione; GSH-Px: glutathione peroxidase; GST: glutathione S-transferase; HbA1c: glycated hemoglobin; HNE: hydroxynonenal; HOMA-IR: homeostatic model assessment for insulin resistance; ITT: insulin tolerance test; MDA: malondialdehyde (a marker of lipid peroxidation); ROS: reactive oxygen species; SOD: superoxide dismutase; TBARS: thiobarbituric acid reactive substances.

**Table 3:** Animal studies that evaluated the effect of blueberry or bilberry consumption in obesity and related-metabolic parameters.

1st Author, Year (Ref)	Sample	Treatment	Follow-Up	Results (BB or BiB vs. Diet-Induced Obesity)
Mouse				
Al-Baghdadi, 2018 [27]	Wild type mice: HSD (*n* = 8) vs. HSD + BB (*n* = 8)	4% of total diet BB (*V. corymbosum* L.) powder	3 months	Improved glucose tolerance (GTT); ↓ BW and *Lep* expression in AT;
De Furia, 2009 [29]	Male C57Bl/6J mice: HFD (*n* = 8) vs. HFD + BB (*n* = 8)	4% wt:wt freeze-dried BB powder (*V. ashei* and *V. corymbosum* 1:1)	8 weeks	↔ No differences in BW, FM, insulin, and *AdipoQ*, *Lep*, *Ccl2 (Mcp1)*, *Il6,* and *iNos* expressions in AT; ↓ blood glucose, AUC_glucose_ GTT, recruitment of pro-inflammatory M1 macrophages (*Cd11c+*/*Mgl-1-*) in AT, *Tnf* and *Il10* expressions in AT, adipocyte death, and IR; ↑ *Gsh-Px* expression in AT;
Elks, 2015 [24]	4-weeks female C57Bl/6J mice with VCB-induced menopause: HFD + VCB (*n* = 6) vs. HFD + VCB + BB (*n* = 8)	4% wt/wt BB powder (*V*. *ashei*)	12 weeks	↔ No differences in BW, FM, FFM, and adiponectin; ↓ hepatic steatosis and TGs (serum and liver), fasting blood glucose, and AUC_glucose_ GTT; ↑ expression of hepatic fatty-acid oxidation-related genes (*Cs*, *Hadha*, and *Cd36*);
Heyman, 2014 [34]	Male C57BL/6J mice: HFD (*n* = 12) vs. HFD + BiB (*n* = 12)	20% wt:wt freeze-dried BiB powder (*V*. *myrtillus*)	13 weeks	↔ No differences in FFM, FPG, TC, LDL, HDL, TGs, and NEFAs (serum); ↓ BW, FM, IR, insulin, Pai-1, ALT, and hepatic cholesterol and TGs;
Lewis, 2018 [50]	Male C57BL/6 mice: HFD (*n* = 9–11) vs. HFD + BB (*n* = 9–11)	4% (wt/wt) freeze-dried BB (*V*. *ashei* and *V. corymbosum* 1:1)	8 or 12 weeks	↑ BW, FM, and T cell proliferation;
Liu, 2019 [20]	Male C57BL/6 mice: HFD (*n* = 5) vs. HFD + BB (*n* = 5)	4% (wt/wt) non-specified freeze-dried BB	14 weeks	↔ No differences in BW and fasting blood glucose; ↓ β-cell expansion, AUC_glucose_ GTT, insulin, and IR; ↑ β-cell survival;
Morissette, 2020 [48]	C57BL/6 male mice: HFHS diet (*n* = 13) vs. HFHS + BB (*n* = 14)	4% (wt/wt) freeze-dried BB powder (*V. ashei* and *V. corymbosum* 1:1)	12 weeks	↔ No differences in BW, FM, FFM, fasting blood glucose, AUC_glucose_ GTT, insulin, IR, hepatic TGs, TC, AST and ALT, TBARS * and *Il2*, *Il6*, *Ifnγ*, *Tnf, Mcp1*, and *Rantes* expressions in AT;
Mykkänen, 2012 [36]	Male C57BL/6 mice: HFD (*n* = 6) vs. HFD + BB (*n* = 6)	5% (wt/wt) freeze-dried powder (*V. myrtillus*)	12 weeks	↔ No differences in BW, weight gain, fasting blood glucose, FFAs, and systolic BP;
Mykkänen, 2014 [51]	Male C57BL mice: HFD (*n* = 20–27) vs. HFD + 5% BiB (*n* = 20–27) vs. HFD + 10% BiB (*n* = 20–27)	5% and 10% (wt/wt) freeze-dried BiB powder (*V. myrtillus*)	12 weeks	↔ No differences in BW, FM, serum adiponectin, leptin, TC, AUC_glucose_ GTT, IR, Tnf, Il1b, Il12, Il7, Il6, and Gm-Csf; ↓ blood glucose, serum resistin and Mcp1 (5 and 10%), Δ-BW (10%), type-1 pro-inflammatory responsiveness (↓ NKT, Th1, Th1/Th2, and Ifnγ-producing T cells—10% BiB), and systolic BP;
Prior, 2008 [44]	Male C57BL/6J mice: HFD (*n* = 12) vs. HFD + BB (*n* = 12)	10% (wt/wt) non-specified freeze-dried whole BB	92 days	↔ No differences in fasting blood glucose and AUC_glucose_ GTT; ↑ BW, FM, and FFM;
Prior, 2010 [37]	Male C57BL/6J mice: HFD vs. HFD + BB	10% (wt/wt) non-specified BB juice	72 days	↔ No differences in BW, weight gain, FFM, FM, TC, TGs, and glucose, insulin, and IR; ↓ serum leptin levels;
Skates, 2018 [45]	Male C57BL/6J mice: HFD (*n* = 12) vs. HFD + BB (*n* = 8 per group)	Non specified freeze-dried whole BB powder (normalized to 400 µg/g total anthocyanins)	12 weeks	↔ BW, *AdipoQ*, *Fasn*, *Cpt1a*, *Pparg*, and *Ppargc1a* expressions (AT); ↓ *Lep* expression (AT) and FM; ↑ FFM, VO_2_ and energy expenditure;
Rats				
Brader, 2013 [23]	Male Zucker diabetic fatty rats: Control (*n* = 12) vs. BiB diet (*n* = 12)	15g of standard chow substituted with freeze-dried BiB (*V. myrtillus*)	8 weeks	↔ No differences in BW, BP, TGs (plasma and liver), FFAs, HbA1c, FPG, AUC_glucoce_ GTT, insulin, adiponectin, and IR; ↓ whole blood glucose, TC, HDL, LDL, and *Glut2*, *Irs1*, *Jnk1*, *Lxr-α*, and *Gfat* expressions (liver); ↑ *Glut4* expression (AT);
Khanal, 2012 [49]	Male Sprague Dawley rats: HFrD (*n* = 6) vs. HFrD + 1.5% BB pomace (*n* = 6 + 6) vs. HFrD + 3% BB pomace (*n* = 6 + 6)	Extruded (*n* = 6) and unextruded (*n* = 6) pomace from non-specified freeze-dried BB	8 weeks	↔ No differences in BW, FM, FPG and leptin, and AUC_glucose_ GTT, ↓ TGs, insulin and TC, and IR (3%);
Lee, 2018 [28]	Male Wistar rats: HFD (*n* = 8) vs. HFD + BB (*n* = 8)	10% (wt/wt) freeze-dried BB (*V*. *ashei* + *V*. *corymbosum* 1:1)	8 weeks	↔ No differences in BW, FM, FPG and insulin, AUC_glucose_ GTT, and *Il6* expression in AT; ↓ *Tnf*, *Il1B*, and *Cd11d* expressions (AT), phospho to total Nf-kb p65 (AT), and MDA ** (liver); ↑ *Pparα* and *Pparδ* expressions (AT); improvement of insulin sensitivity markers;
Liu, 2019 [35]	Male Sprague Dawley rats: HFD (*n* = 8) vs. HFD + BiB (*n* = 8)	7% of the dietary fiber (dry weight basis) of *V*. *mirtyllus*	8 weeks	↔ No differences in TGs, FFAs, and hepatic *Pparα*, *Pparγ*, *Fasn*, *Fabp5*, and *Cpt1α* expressions; ↓ BW, hepatic steatosis, and TC; ↑ iBAT mass and *Ucp1* expression in iBAT;
Nair, 2014 [32]	Obese Zucker rats (OZR): Control diet (*n* = 7) vs. BB diet (*n* = 7)	2% non-specified freeze-dried BB	15 weeks	↔ No differences in BW; ↓ mean BP, AUC_glucose_ GTT, kidney expressions of *Il1β*, *Il18*, *TgfB*, *Tlr4* and p38/Mapk phosphorylation, Nf-kb activity, total ROS, superoxide and peroxynitrite ***, UAE levels, glomerular sclerosis, and interstitial nephritis; ↑ eGFR, Sod and catalase;
Nunes, 2021 [33]	Male Wistar rats: HSuHF + BB juice (*n* = 10) vs. HSuHF (*n* = 10) vs. CTRL (*n* = 8)	25 g/kg BW/day of BB juice (BB blended with 35% sucrose solution) (*V*. *corymbosum)*		↔ No differences in BW, Δ-BW, fasting insulin and glucose; *Il10*, *Adipor1 e 2*, *Tnf*, *Fasn*, *Ifnγ*, *Il1β*, *Nf-kb, Irs1*, *Insr*, and *Stat3* expressions; ↓ fed insulin and glucose, AUC_glucose_ GTT, IR, serum and hepatic TGs, MDA and MDA/total antioxidant status ratio ****, hepatic steatosis; ↑ hepatic GSH;
Seymour, 2011 [26]	Male OZR: HFD (*n* = 12) vs. HFD + BB (*n* = 12)	2% (wt/wt) freeze-dried whole BB powder (*V*. *ashei* + *V*. *corymbosum*)	90 days	↔ No differences in BW, FFM, FM, fasting blood glucose, CT, FFAs, HDL, Tnf, and Il6; ↓ TGs, insulin, IR, AUC_glucose_ GTT; ↑ *Ppara* (AT)*, Ppargc1a*, *Glut4*, *Irs1*, and *Fasn* (AT and skeletal muscle), and *Pparγ and Ucp3* (skeletal muscle) expressions;
Vendrame, 2013 [30]	Male OZR: Control diet (*n* = 10) vs. BB diet (*n* = 10)	8% wt/wt freeze-dried BB powder (*V*. *angustifolium*)	8 weeks	↔ No differences in BW and weight gain; ↓ Tnf and Il6 (plasma, liver, and AT), CRP (plasma and liver), and Nf-kb levels (AT and liver); ↑ plasma adiponectin;
Vendrame, 2014 [21]	Male OZR: Control diet (*n* = 10) vs. BB diet (*n* = 10)	8% wt/wt freeze-dried BB powder (*V*. *angustifolium*)	8 weeks	↔ No differences in BW, HDL, *Ppara*, *Pparg*, and *Abca1* expressions (liver); ↓ TGs, TC, and *Srebp1*, and *Fasn* expressions (AT and liver), ↑ *Pparγ*, *Pparα*, and *Abca1* expressions (AT);
Vendrame, 2015 [38]	Male OZR: Control diet (*n* = 10) vs. BB diet (*n* = 10)	8% wt/wt non-specified freeze-dried BB powder	8 weeks	↔ No differences in BW, IR, fasting blood glucose and insulin, and *Glut4* expression (liver/AT); ↓ HbA1c, Rbp4 and resistin (plasma), *Rbp4* (liver and AT) and *resistin* (liver) expressions;
Guinea pig				
Çoban, 2013 [31]	Male Dankin Hartley guinea pigs: HCD (*n* = 6) vs. HCD + BB (*n* = 6)	8% wt/wt whole BB (*V. corymbosum* L.) powder	75 days	↔ HDL and TGs (serum), and GSH, GSH-Px, and SOD (liver); ↓ TC (serum, liver and aorta), LDL, ALT and AST (serum), and TGs and MDA **** (liver); ↑ GST (liver);

* Lipid peroxidation was assessed using a thiobarbituric acid reactive substances (TBARS) assay kit (R&D Systems); ** MDA (malondialdehyde; a marker of lipid peroxidation) was measured by using an ELISA kit (Oxford Biomedical Research); *** Total reactive oxygen species (ROS), superoxide, and peroxynitrite production rates were measure in pieces of kidney cortex using electron paramagnetic resonance spectroscopy—EPR (Benchtop EPR spectrophotometer e-scan R—Noxygen Science Transfer). Total ROS represents superoxide, hydrogen peroxide, and hydroxyl radical, with other species in minimal amounts. **** MDA levels were measured through TBARS colorimetric test. Total antioxidant status (TAS) was estimated through the ferric reducing antioxidant potential (FRAP) assay and measured in a spectrometer. ↓ Decreased values; ↑ Increased values; ALT: alanine aminotransferase; AST: aspartate aminotransferase; AT: adipose tissue; AUC_glucose_ GTT: area under the curve of glucose in the glucose tolerance test; BB: blueberry; BiB: bilberry; BP: blood pressure; BW: body weight; CRP: *C*-reactive protein; FFAs: free-fat acids; FFM: fat-free mass; FM: fat mass; FPG: fasting plasma glucose; eGFR: estimated glomerular filtration rate; GSH: glutathione; GSH-Px: glutathione peroxidase; GST: glutathione *S*-transferase; HbA1c: glycated hemoglobin; HCD: high-cholesterol diet; HFD: high-fat diet; HFHS: high-fat, high-sucrose diet; HFrD: high fructose diet; HSD: high-sugar diet; HSuHF: high sucrose diet combined with high-fat diet; iBAT: interscapular brown adipose tissue; IR: insulin resistance; LFD: low fat diet; LZR: lean Zucker rat; NEFAs: non-esterified fatty acids; NKT: natural killer T cells; OLETF: Otsuka Long-Evans Tokushima fatty rats; OZR: obese Zucker rat; SOD: superoxide dismutase; TC: total cholesterol; TGs: triglycerides; UAE: urinary albumin excretion; *V*.: *Vaccinium*.

## Supplementary Materials

The following supporting information can be downloaded at: https://www.mdpi.com/article/10.3390/metabo13010019/s1. Table S1: PRISMA checklist, Table S2: Clinical characteristics of patients and other relevant information of the human studies.

## Abbreviations

ALT	Alanine aminotransferase
AST	Aspartate aminotransferase
AUC	Area under the curve
BB	Blueberry
BiB	Bilberry
BW	Body weight
BMI	Body mass index
BP	Blood pressure
CCL2	C-C motif chemokine ligand 2
MCP1	Monocyte chemoattractant protein-1
Δ-BW	Delta body weight
FFM	Fat-free mass
FM	Fat mass
FPG	Fasting plasma glucose
GLUT	Glucose transporter
GSH	Glutathione
GSH-Px	Glutathione peroxidase
GTT	Glucose tolerance test
HbA1c	Glycated hemoglobin
HFD	High-fat diet
HNE	Hydroxynonenal
hs-CRP	High sensitivity *C*-reactive protein
IL	Interleukin
IR	Insulin resistance
IRS1	Insulin receptor substrate 1
MDA	Malondialdehyde
MetS	Metabolic syndrome
NAFLD	Non-alcoholic fatty liver disease
NF-κB	Nuclear factor-kappa B
PPARγ	Peroxisome proliferator-activated receptor-γ
RBP4	Retinol-binding protein 4
ROS	Reactive oxygen species
SOD	Superoxide dismutase
Th	T helper
TG	Triglycerides
T2DM	Type 2 diabetes mellitus
TNF	Tumor necrosis factor
TBARS	Thiobarbituric acid reactive substances
V.	Vaccinium

## References

[1] World Health Organization Obesity. https://www.who.int/health-topics/obesity#tab=tab_1.

[2] Lobstein T., Neveux H.B., Cavalcanti O.B., Barquera S., Louise Baur V.B., Buse K., Dietz B., French A., Leach R.J., Opzeeland B. (2022). World Obesity Atlas 2022.

[3] Apovian C.M. (2016). Obesity: Definition, comorbidities, causes, and burden. Am. J. Manag. Care.

[4] Arroyo-Johnson C., Mincey K.D. (2016). Obesity Epidemiology Worldwide. Gastroenterol. Clin. N. Am..

[5] Ladabaum U., Mannalithara A., Myer P.A., Singh G. (2014). Obesity, abdominal obesity, physical activity, and caloric intake in US adults: 1988 to 2010. Am. J. Med..

[6] Ng S.W., Popkin B.M. (2012). Time use and physical activity: A shift away from movement across the globe. Obes. Rev..

[7] Magliano D.J., Boyko E.J., Balkau B., Barengo N., Barr E., Basit A., Bhata D., Bommer C., Booth G., Cariou B. (2021). IDF Diabetes Atlas 2021.

[8] Vona R., Gambardella L., Cittadini C., Straface E., Pietraforte D. (2019). Biomarkers of Oxidative Stress in Metabolic Syndrome and Associated Diseases. Oxid. Med. Cell. Longev..

[9] Tian C., Hao L., Yi W., Ding S., Xu F. (2020). Polyphenols, Oxidative Stress, and Metabolic Syndrome. Oxid. Med. Cell. Longev..

[10] McMurray F., Patten D.A., Harper M.E. (2016). Reactive Oxygen Species and Oxidative Stress in Obesity-Recent Findings and Empirical Approaches. Obesity.

[11] Rani V., Deep G., Singh R.K., Palle K., Yadav U.C. (2016). Oxidative stress and metabolic disorders: Pathogenesis and therapeutic strategies. Life Sci..

[12] Guo X., Yang B., Tan J., Jiang J., Li D. (2016). Associations of dietary intakes of anthocyanins and berry fruits with risk of type 2 diabetes mellitus: A systematic review and meta-analysis of prospective cohort studies. Eur. J. Clin. Nutr..

[13] Jennings A., Welch A.A., Spector T., Macgregor A., Cassidy A. (2014). Intakes of anthocyanins and flavones are associated with biomarkers of insulin resistance and inflammation in women. J. Nutr..

[14] Kouki R., Schwab U., Hassinen M., Komulainen P., Heikkila H., Lakka T.A., Rauramaa R. (2011). Food consumption, nutrient intake and the risk of having metabolic syndrome: The DR’s EXTRA Study. Eur. J. Clin. Nutr..

[15] Koponen J.M., Happonen A.M., Mattila P.H., Torronen A.R. (2007). Contents of anthocyanins and ellagitannins in selected foods consumed in Finland. J. Agric. Food Chem..

[16] Basu A., Du M., Leyva M.J., Sanchez K., Betts N.M., Wu M., Aston C.E., Lyons T.J. (2010). Blueberries decrease cardiovascular risk factors in obese men and women with metabolic syndrome. J. Nutr..

[17] Stull A.J., Cash K.C., Johnson W.D., Champagne C.M., Cefalu W.T. (2010). Bioactives in blueberries improve insulin sensitivity in obese, insulin-resistant men and women. J. Nutr..

[18] Curtis P.J., van der Velpen V., Berends L., Jennings A., Feelisch M., Umpleby A.M., Evans M., Fernandez B.O., Meiss M.S., Minnion M. (2019). Blueberries improve biomarkers of cardiometabolic function in participants with metabolic syndrome-results from a 6-month, double-blind, randomized controlled trial. Am. J. Clin. Nutr..

[19] Kolehmainen M., Mykkänen O., Kirjavainen P.V., Leppänen T., Moilanen E., Adriaens M., Laaksonen D.E., Hallikainen M., Puupponen-Pimiä R., Pulkkinen L. (2012). Bilberries reduce low-grade inflammation in individuals with features of metabolic syndrome. Mol. Nutr. Food Res..

[20] Liu W., Mao Y., Schoenborn J., Wang Z., Tang G., Tang X. (2019). Whole blueberry protects pancreatic beta-cells in diet-induced obese mouse. Nutr. Metab..

[21] Vendrame S., Daugherty A., Kristo A.S., Klimis-Zacas D. (2014). Wild blueberry (Vaccinium angustifolium)-enriched diet improves dyslipidaemia and modulates the expression of genes related to lipid metabolism in obese Zucker rats. Br. J. Nutr..

[22] Nair A.R., Mariappan N., Stull A.J., Francis J. (2017). Blueberry supplementation attenuates oxidative stress within monocytes and modulates immune cell levels in adults with metabolic syndrome: A randomized, double-blind, placebo-controlled trial. Food Funct..

[23] Brader L., Overgaard A., Christensen L.P., Jeppesen P.B., Hermansen K. (2013). Polyphenol-rich bilberry ameliorates total cholesterol and LDL-cholesterol when implemented in the diet of Zucker diabetic fatty rats. Rev. Diabet. Stud.: RDS.

[24] Elks C.M., Terrebonne J.D., Ingram D.K., Stephens J.M. (2015). Blueberries improve glucose tolerance without altering body composition in obese postmenopausal mice. Obesity.

[25] Keirsey K.I., Lee S., De La Serre C.B., Fischer J.G. (2016). Blueberry supplementation alters biomarkers of oxidative stress in high-fat fed rats. FASEB J..

[26] Seymour E.M., Tanone I.I., Urcuyo-Llanes D.E., Lewis S.K., Kirakosyan A., Kondoleon M.G., Kaufman P.B., Bolling S.F. (2011). Blueberry intake alters skeletal muscle and adipose tissue peroxisome proliferator-activated receptor activity and reduces insulin resistance in obese rats. J. Med. Food.

[27] Al-Baghdadi R.J.T. (2018). Effects of refined sugar on some endocrine glands functions in mice. J. Glob. Pharma Technol..

[28] Lee S., Keirsey K.I., Kirkland R., Grunewald Z.I., Fischer J.G., de La Serre C.B. (2018). Blueberry Supplementation Influences the Gut Microbiota, Inflammation, and Insulin Resistance in High-Fat-Diet-Fed Rats. J. Nutr..

[29] DeFuria J., Bennett G., Strissel K.J., Perfield Ii J.W., Milbury P.E., Greenberg A.S., Obin M.S. (2009). Dietary blueberry attenuates whole-body insulin resistance in high fat-fed mice by reducing adipocyte death and its inflammatory sequelae. J. Nutr..

[30] Vendrame S., Daugherty A., Kristo A.S., Riso P., Klimis-Zacas D. (2013). Wild blueberry (Vaccinium angustifolium) consumption improves inflammatory status in the obese Zucker rat model of the metabolic syndrome. J. Nutr. Biochem..

[31] Coban J., Evran B., Ozkan F., Cevik A., Dogru-Abbasoglu S., Uysal M. (2013). Effect of blueberry feeding on lipids and oxidative stress in the serum, liver and aorta of guinea pigs fed on a high-cholesterol diet. Biosci. Biotechnol. Biochem..

[32] Nair A.R., Elks C.M., Vila J., Del Piero F., Paulsen D.B., Francis J. (2014). A blueberry-enriched diet improves renal function and reduces oxidative stress in metabolic syndrome animals: Potential mechanism of TLR4-MAPK signaling pathway. PLoS ONE.

[33] Nunes S., Viana S.D., Preguiça I. (2021). Blueberry Counteracts Prediabetes in a Hypercaloric Diet-Induced Rat Model and Rescues Hepatic Mitochondrial Bioenergetics. Nutrients.

[34] Heyman L., Axling U., Blanco N., Sterner O., Holm C., Berger K. (2014). Evaluation of Beneficial Metabolic Effects of Berries in High-Fat Fed C57BL/6J Mice. J. Nutr. Metab..

[35] Liu H.Y., Walden T.B., Cai D., Ahl D., Bertilsson S., Phillipson M., Nyman M., Holm L. (2019). Dietary fiber in bilberry ameliorates pre-obesity events in rats by regulating lipid depot, cecal short-chain fatty acid formation and microbiota composition. Nutrients.

[36] Mykkänen O.T., Kalesnykas G., Adriaens M., Evelo C.T., Törrönen R., Kaarniranta K. (2012). Bilberries potentially alleviate stress-related retinal gene expression induced by a high-fat diet in mice. Mol. Vis..

[37] Prior R.L., Wilkes S.E., Rogers T.R., Khanal R.C., Wu X., Howard L.R. (2010). Purified blueberry anthocyanins and blueberry juice alter development of obesity in mice fed an obesogenic high-fat diet. J. Agric. Food Chem..

[38] Vendrame S., Zhao A., Merrow T., Klimis-Zacas D. (2015). The effects of wild blueberry consumption on plasma markers and gene expression related to glucose metabolism in the obese Zucker rat. J. Med. Food.

[39] Cochrane, Cochrane Handbook for Systematic Reviews of Interventions version 6.2. Higgins, J.; Thomas, J.; Chandler, J.; Cumpston, M.; Li, T.; Page, M.; Welch, V., Eds. Cochrane 2021. www.training.cochrane.org/handbook.

[40] Page M.J., McKenzie J.E., Bossuyt P.M., Boutron I., Hoffmann T.C., Mulrow C.D., Shamseer L., Tetzlaff J.M., Akl E.A., Brennan S.E. (2021). The PRISMA 2020 statement: An updated guideline for reporting systematic reviews. BMJ.

[41] Brondani L.A., Assmann T.S., de Souza B.M., Boucas A.P., Canani L.H., Crispim D. (2014). Meta-analysis reveals the association of common variants in the uncoupling protein (UCP) 1-3 genes with body mass index variability. PLoS ONE.

[42] Brondani L.A., de Souza B.M., Assmann T.S., Boucas A.P., Bauer A.C., Canani L.H., Crispim D. (2014). Association of the UCP polymorphisms with susceptibility to obesity: Case-control study and meta-analysis. Mol. Biol. Rep..

[43] Curtis P.J., Berends L., van der Velpen V., Jennings A., Haag L., Chandra P., Kay C.D., Rimm E.B., Cassidy A. (2022). Blueberry anthocyanin intake attenuates the postprandial cardiometabolic effect of an energy-dense food challenge: Results from a double blind, randomized controlled trial in metabolic syndrome participants. Clin. Nutr..

[44] Prior R.L., Wu X., Gu L., Hager T.J., Hager A., Howard L.R. (2008). Whole berries versus berry anthocyanins: Interactions with dietary fat levels in the C57BL/6J mouse model of obesity. J. Agric. Food Chem..

[45] Skates E., Overall J., DeZego K., Wilson M., Esposito D., Lila M.A., Komarnytsky S. (2018). Berries containing anthocyanins with enhanced methylation profiles are more effective at ameliorating high fat diet-induced metabolic damage. Food Chem. Toxicol..

[46] Stote K.S., Wilson M.M., Hallenbeck D., Thomas K., Rourke J.M., Sweeney M.I., Gottschall-Pass K.T., Gosmanov A.R. (2020). Effect of blueberry consumption on cardiometabolic health parameters in men with type 2 diabetes: An 8-week, double-blind, randomized, placebo-controlled trial. Curr. Dev. Nutr..

[47] Stull A.J., Cash K.C., Champagne C.M., Gupta A.K., Boston R., Beyl R.A., Johnson W.D., Cefalu W.T. (2015). Blueberries improve endothelial function, but not blood pressure, in adults with metabolic syndrome: A randomized, double-blind, placebo-controlled clinical trial. Nutrients.

[48] Morissette A., Kropp C., Songpadith J.P., Junges Moreira R., Costa J., Mariné Casadó R., Pilon G., Varin T.V., Dudonné S., Boutekrabt L. (2020). Blueberry proanthocyanidins and anthocyanins improve metabolic health through a gut microbiota-dependent mechanism in diet-induced obese mice. Am. J. Physiology. Endocrinol. Metab..

[49] Khanal R.C., Howard L.R., Wilkes S.E., Rogers T.J., Prior R.L. (2012). Effect of dietary blueberry pomace on selected metabolic factors associated with high fructose feeding in growing Sprague-Dawley rats. J. Med. Food.

[50] Lewis E.D., Ren Z., DeFuria J., Obin M.S., Meydani S.N., Wu D. (2018). Dietary supplementation with blueberry partially restores T-cell-mediated function in high-fat-diet-induced obese mice. Br. J. Nutr..

[51] Mykkänen O.T., Huotari A., Herzig K.H., Dunlop T.W., Mykkänen H., Kirjavainen P.V. (2014). Wild blueberries (Vaccinium myrtillus) alleviate inflammation and hypertension associated with developing obesity in mice fed with a high-fat diet. PLoS ONE.

[52] Stull A.J., Beyl R.A. (2016). Blueberries improve whole-body insulin action and alter the development of obesity in high-fat fed mice. FASEB J..

[53] Vendrame S., Kristo A.S., Schuschke D.A., Klimis-Zacas D. (2014). Wild blueberry consumption affects aortic vascular function in the obese Zucker rat. Appl. Physiol. Nutr. Metab. = Physiol. Appl. Nutr. Et Metab..

[54] Olas B. (2018). Berry Phenolic Antioxidants—Implications for Human Health?. Front. Pharmacol..

[55] Seeram N.P. (2008). Berry fruits: Compositional elements, biochemical activities, and the impact of their intake on human health, performance, and disease. J. Agric. Food Chem..

[56] Howell A.B. (2007). Bioactive compounds in cranberries and their role in prevention of urinary tract infections. Mol. Nutr. Food Res..

[57] Skrovankova S., Sumczynski D., Mlcek J., Jurikova T., Sochor J. (2015). Bioactive Compounds and Antioxidant Activity in Different Types of Berries. Int. J. Mol. Sci..

[58] Lee Y.M., Yoon Y., Yoon H., Park H.M., Song S., Yeum K.J. (2017). Dietary Anthocyanins against Obesity and Inflammation. Nutrients.

[59] Cladis D.P., Li S., Reddivari L., Cox A., Ferruzzi M.G., Weaver C.M. (2020). A 90 day oral toxicity study of blueberry polyphenols in ovariectomized sprague-dawley rats. Food Chem. Toxicol..

[60] Lin Z., Fischer J., Wicker L. (2016). Intermolecular binding of blueberry pectin-rich fractions and anthocyanin. Food Chem..

[61] Eberhardt M.V., Lee C.Y., Liu R.H. (2000). Antioxidant activity of fresh apples. Nature.

[62] Nayak B., Liu R.H., Tang J. (2015). Effect of processing on phenolic antioxidants of fruits, vegetables, and grains—A review. Crit. Rev. Food Sci. Nutr..

[63] Sellappan S., Akoh C.C., Krewer G. (2002). Phenolic compounds and antioxidant capacity of Georgia-grown blueberries and blackberries. J. Agric. Food Chem..

[64] Ahmet I., Spangler E., Shukitt-Hale B., Juhaszova M., Sollott S.J., Joseph J.A., Ingram D.K., Talan M. (2009). Blueberry-enriched diet protects rat heart from ischemic damage. PLoS ONE.

[65] Fiorentino T.V., Prioletta A., Zuo P., Folli F. (2013). Hyperglycemia-induced oxidative stress and its role in diabetes mellitus related cardiovascular diseases. Curr. Pharm. Des..

[66] Sobolev A.P., Ciampa A., Ingallina C., Mannina L., Capitani D., Ernesti I., Maggi E., Businaro R., Del Ben M., Engel P. (2019). Blueberry-based meals for obese patients with metabolic syndrome: A multidisciplinary metabolomic pilot study. Metabolites.

[67] Haddad J.J., Abdel-Karim N.E. (2011). NF-kappaB cellular and molecular regulatory mechanisms and pathways: Therapeutic pattern or pseudoregulation?. Cell. Immunol..

[68] Aboonabi A., Aboonabi A. (2020). Anthocyanins reduce inflammation and improve glucose and lipid metabolism associated with inhibiting nuclear factor-kappaB activation and increasing PPAR-gamma gene expression in metabolic syndrome subjects. Free Radic. Biol. Med..

[69] Huang W.Y., Liu Y.M., Wang J., Wang X.N., Li C.Y. (2014). Anti-inflammatory effect of the blueberry anthocyanins malvidin-3-glucoside and malvidin-3-galactoside in endothelial cells. Molecules.

[70] Lee S.G., Kim B., Yang Y., Pham T.X., Park Y.K., Manatou J., Koo S.I., Chun O.K., Lee J.Y. (2014). Berry anthocyanins suppress the expression and secretion of proinflammatory mediators in macrophages by inhibiting nuclear translocation of NF-kappaB independent of NRF2-mediated mechanism. J. Nutr. Biochem..

[71] Paixao J., Dinis T.C., Almeida L.M. (2012). Malvidin-3-glucoside protects endothelial cells up-regulating endothelial NO synthase and inhibiting peroxynitrite-induced NF-kB activation. Chem. Biol. Interact..

[72] Karlsen A., Paur I., Bohn S.K., Sakhi A.K., Borge G.I., Serafini M., Erlund I., Laake P., Tonstad S., Blomhoff R. (2010). Bilberry juice modulates plasma concentration of NF-kappaB related inflammatory markers in subjects at increased risk of CVD. Eur. J. Nutr..

[73] Karlsen A., Retterstol L., Laake P., Paur I., Bohn S.K., Sandvik L., Blomhoff R. (2007). Anthocyanins inhibit nuclear factor-kappaB activation in monocytes and reduce plasma concentrations of pro-inflammatory mediators in healthy adults. J. Nutr..

[74] Incalza M.A., D’Oria R., Natalicchio A., Perrini S., Laviola L., Giorgino F. (2018). Oxidative stress and reactive oxygen species in endothelial dysfunction associated with cardiovascular and metabolic diseases. Vascul. Pharmacol..

[75] Xu J.W., Ikeda K., Yamori Y. (2004). Cyanidin-3-glucoside regulates phosphorylation of endothelial nitric oxide synthase. FEBS Lett..

[76] Xu J.W., Ikeda K., Yamori Y. (2004). Upregulation of endothelial nitric oxide synthase by cyanidin-3-glucoside, a typical anthocyanin pigment. Hypertension.

[77] Lazze M.C., Pizzala R., Perucca P., Cazzalini O., Savio M., Forti L., Vannini V., Bianchi L. (2006). Anthocyanidins decrease endothelin-1 production and increase endothelial nitric oxide synthase in human endothelial cells. Mol. Nutr. Food Res..

[78] Rozanska D., Regulska-Ilow B. (2018). The significance of anthocyanins in the prevention and treatment of type 2 diabetes. Adv. Clin. Exp. Med..

[79] AlShaalan R., Aljiffry M., Al-Busafi S., Metrakos P., Hassanain M. (2015). Nonalcoholic fatty liver disease: Noninvasive methods of diagnosing hepatic steatosis. Saudi J. Gastroenterol..

[80] Wei X., Wang D., Yang Y., Xia M., Li D., Li G., Zhu Y., Xiao Y., Ling W. (2011). Cyanidin-3-O-beta-glucoside improves obesity and triglyceride metabolism in KK-Ay mice by regulating lipoprotein lipase activity. J. Sci. Food Agric..

[81] Tsuda T., Ueno Y., Kojo H., Yoshikawa T., Osawa T. (2005). Gene expression profile of isolated rat adipocytes treated with anthocyanins. Biochim. Biophys. Acta.

[82] Xia M., Hou M., Zhu H., Ma J., Tang Z., Wang Q., Li Y., Chi D., Yu X., Zhao T. (2005). Anthocyanins induce cholesterol efflux from mouse peritoneal macrophages: The role of the peroxisome proliferator-activated receptor gamma-liver X receptor alpha-ABCA1 pathway. J. Biol. Chem..

