# Supplementary material for: Blueberry Consumption and Changes in Obesity and Diabetes Mellitus Outcomes: A Systematic Review
Source: Metabolites. 2022 Dec 22;13(1):19. doi: 10.3390/metabo13010019 (PMC9861336; doi:10.3390/metabo13010019)
Supplement: Supplementary file 1 [file metabolites-13-00019-s001.zip › Table S2.pdf]

Table S2. Clinical characteristics of patients and other relevant information of the human studies.

1st author, year [ref]	Sample	Characteristic Placebo group	Characteristic BB or BiB group	Other relevant information
Basu, 2010 [16]	48 subjects with MetS: BB group (*n*=25) *vs*. controls (*n*=23)	Age: 48.0 ± 3.3<br>BMI: 37.5 ± 3.0<br>Male (%): 9.5	Age: 51.5 ± 3.0<br>BMI: 38.1 ± 1.5<br>Male (%): 8	All patients had obesity;<br>Exclusion criteria: use of medication for any chronic disease; presence of liver, renal, or thyroid disorders or anemia; consumption of antioxidants, fish oil supplements or alcohol on a regular basis; current smokers; pregnancy or lactating women.<br>ROS measurement: MDA and HNE were measured using colorimetric assays (LPO-586, Oxis Health Products).
Curtis, 2019 [18]	115 subjects with MetS: 150 g BB (*n*=37), 75 g BB (*n*=39) *vs*. controls (*n*=39)	Age: 62.9 ± 8.1<br>BMI: 31.1 ± 3.0<br>Male (%): 66.7	75g/150g:<br>Age: 62.6 ± 7.2/63.0 ± 5.9<br>BMI: 31.2 ± 2.6/31.3 ± 3.4<br>Male (%): 71.8/64.9	Patients with overweight or obesity; 24.3% used antihypertensive and 38.3% lipid-lowering medications (total sample; = between groups).<br>Exclusion criteria: smokers; presence of DM, vascular disease, untreated hypertension, cancer, or digestive, hepatic or renal disorders; use of hypoglycemic, vasodilator, or hormone-replacement medications.<br>Endothelial function: 3-lead electrocardiogram gated brachial artery flow-mediated dilation (% FMD) and arterial stiffness.*
Curtis, 2022 [43]	45 subjects with MetS: BB (*n*=23) *vs*. controls (*n*=22)	Age: 63.2 ± 8.8<br>BMI: 31.5 ± 2.9<br>Male (%): 59.1	Age: 63.6 ± 6.0<br>BMI: 31.3 ± 3.4<br>Male (%): 69.6	Patients with overweight or obesity; 37.8% used antihypertensive and 44.4% lipid-lowering medications (total sample; % between groups).<br>Exclusion criteria: presence of DM, vascular disease or cancer; smokers; use of hormone replacement therapy, hypoglycemic or vasodilator medications.<br>Endothelial function: 3-lead electrocardiogram gated brachial artery FMD and arterial stiffness.*
Kolehmainen, 2012 [19]	27 subjects with MetS: BiB diet (*n*=15) *vs*. controls (*n*=12)	Age: 50 ± 7<br>BMI: 32.9 ± 3.4<br>Male (%): 25	Age: 53 ± 6<br>BMI: 31.4 ± 4.7<br>Male (%): 50	Patients with overweight or obesity;<br>Exclusion criteria: Not described.
Nair, 2017 [22]	27 subjects with MetS: BB (*n*=15) *vs*. controls (*n*=12)	Age: 59 ± 3<br>BMI: 35.2 ± 1.4<br>Male (%): 16.7	Age: 55 ± 2<br>BMI: 34.2 ± 0.8<br>Male (%): 13.3	All participants abstained from using nonprescription drugs, vitamins, dietary and herbal supplements two weeks prior to the start of the study;<br>Exclusion criteria: smokers; presence of DM, liver, heart or kidney diseases.<br>ROS measurement: total ROS (superoxide, hydrogen peroxide, hydroxyl radical, and other species) and superoxide production rates were measured in whole blood and monocytes using electron paramagnetic resonance spectroscopy (BenchTop EPR spectrophotometer e-scan R – Noxygen Science Transfer). 1-Hydroxy-3 methoxycarbonyl2,2,5,5-tetramethylpyrollidine was used to measure total ROS and superoxide.
Stote, 2020 [46]	52 men with T2DM: BB (*n*=26) *vs*. placebo (*n*=26)	Age: 66.7 ± 1.1<br>BMI: 34.0 ± 0.9	Age: 67.1 ± 1.1<br>BMI: 34.2 ± 0.7	Patients with a medical history of T2DM for ≥ 6 months, HbA1c ≥ 6.5, and BMI ≥ 25 kg/m^2. Antihypertensive medications: 81% of the placebo group and 92% of the BB group; lipid-lowering medications: 88% placebo group and 65% of BB group.

				<u>Exclusion criteria</u>: use of insulin; presence of chronic kidney disease, liver cirrhosis, gastrointestinal or pancreatic diseases, or malabsorption syndromes; weight loss ≥ 10% BW within the past 12 months; participation in a heavy exercise program or initiation of exercise during program; heavy smokers; known allergy or adverse reaction to BB.
Stull, 2010 [17]	32 individuals with obesity and IR: BB (*n*=15) *vs*. controls (*n*=17)	Age: 49 ± 3<br>BMI: 38 ± 0.9<br>Male (%): 17.6	Age: 54 ± 3<br>BMI: 36.8 ± 0.9<br>Male (%): 13.3	BMI between 32 and 45 kg/m^2 and with IR;<br><u>Exclusion criteria</u>: presence of DM, thyroid, liver, renal, or cardiovascular diseases; hypoglycemic medications; drug or alcohol abuse; allergy to BB; consumption of berries, grapes, and wine >3 times/week; fluctuations in BW;
Stull, 2015 [47]	44 individuals with MetS: BB (*n*=23) *vs*. control (*n*=21)			Majority of participants were on antihypertensive medications; all participants abstained from using nonprescription drugs, vitamins, dietary, and herbal supplements two weeks prior to the start of the study;<br><u>Exclusion criteria</u>: presence of DM; liver, kidney, or heart diseases; smokers.

* FMD = 3-lead electrocardiogram gated brachial artery FMD was assessed by ultrasound (Philips iE33; 11–13 MHz linear transducer) as follows: 1 min baseline, 5 min reactive hyperemia (via 220 mmHg sphygmomanometric cuff inflation), 5 min postocclusion (following cuff deflation). Image acquisition and automated edge-detection analysis were performed with commercial software (Vascular Imager and Brachial Analyzer version 5, respectively; Medical Imaging Applications LLC) and % FMD was calculated as (diametermax − diameterbaseline)/diameterbaseline × 100. Arterial stiffness (standardized to a heart rate of 75) was assessed via the AIx (a measure of the wave reflection to arterial pressure waveforms; Vicorder, Smart Medical) with a target mean of < 10% CV.

HbA1c: glycated hemoglobin; BB: blueberry; BiB: bilberry; BMI: body mass index; BW: body weight; DM: diabetes mellitus; HNE: hydroxynonenal; MDA: malondialdehyde; MetS: metabolic syndrome; ROS: reactive oxygen species; T2DM: type 2 diabetes mellitus.
